# Migalastat improves diarrhea in patients with Fabry disease: clinical-biomarker correlations from the phase 3 FACETS trial

**DOI:** 10.1186/s13023-018-0813-7

**Published:** 2018-04-27

**Authors:** Raphael Schiffmann, Daniel G. Bichet, Ana Jovanovic, Derralynn A. Hughes, Roberto Giugliani, Ulla Feldt-Rasmussen, Suma P. Shankar, Laura Barisoni, Robert B. Colvin, J. Charles Jennette, Fred Holdbrook, Andrew Mulberg, Jeffrey P. Castelli, Nina Skuban, Jay A. Barth, Kathleen Nicholls

**Affiliations:** 1 Baylor Scott & White Research Institute, Dallas, TX USA; 2 Hôpital du Sacré-Coeur, University of Montreal, Montreal, Quebec Canada; 3 Salford Royal Foundation Trust, Manchester, Greater Manchester, UK; 4 NHS Foundation Trust, Royal Free Hospital, London, UK; 5 Medical Genetics Service, HCPA/UFRGS, Porto Alegre, Brazil; 6 Rigshospitalet, University of Copenhagen, Copenhagen, Denmark; 7 Emory University School of Medicine, Atlanta, GA USA; 8 Miller School of Medicine, University of Miami, Miami, FL USA; 9 Massachusetts General Hospital, Harvard Medical School, Boston, MA USA; 10 School of Medicine, University of North Carolina at Chapel Hill, Chapel Hill, NC USA; 11 Amicus Therapeutics, Inc., Cranbury, NJ USA; 12 Department of Nephrology, Royal Melbourne Hospital, Parkville, VIC Australia; 13 Present Address: UC Davis MIND Institute, Sacramento, CA USA; 14 Institute of Metabolic Disease, 3812 Elm Street, Dallas, TX 75226 USA

**Keywords:** Amenable mutation, Diarrhea, Fabry disease, Gastrointestinal, Globotriaosylceramide, GSRS, Lyso-Gb_3_, Migalastat, Pharmacological chaperone

## Abstract

**Background:**

Fabry disease is frequently characterized by gastrointestinal symptoms, including diarrhea. Migalastat is an orally-administered small molecule approved to treat the symptoms of Fabry disease in patients with amenable mutations.

**Methods:**

We evaluated minimal clinically important differences (MCID) in diarrhea based on the corresponding domain of the patient-reported Gastrointestinal Symptom Rating Scale (GSRS) in patients with Fabry disease and amenable mutations (*N* = 50) treated with migalastat 150 mg every other day or placebo during the phase 3 FACETS trial (NCT00925301).

**Results:**

After 6 months, significantly more patients receiving migalastat versus placebo experienced improvement in diarrhea based on a MCID of 0.33 (43% vs 11%; *p* = .02), including the subset with baseline diarrhea (71% vs 20%; *p* = .02). A decline in kidney peritubular capillary globotriaosylceramide inclusions correlated with diarrhea improvement; patients with a reduction > 0.1 were 5.6 times more likely to have an improvement in diarrhea than those without (*p* = .031).

**Conclusions:**

Migalastat was associated with a clinically meaningful improvement in diarrhea in patients with Fabry disease and amenable mutations. Reductions in kidney globotriaosylceramide may be a useful surrogate endpoint to predict clinical benefit with migalastat in patients with Fabry disease.

**Trial registration:**

NCT00925301; June 19, 2009.

## Background

Fabry disease is a rare, progressive, life-threatening X-linked lysosomal storage disorder, affecting males and females, with an estimated prevalence of 1:117,000 to 1:40,000 [1, 2]. Mutations in the *GLA* gene can lead to a deficiency of the lysosomal enzyme α-galactosidase A, which in turn results in an accumulation of glycosphingolipids, including globotriaosylceramide (GL-3) and plasma globotriaosylsphingosine (lyso-Gb_3_), and subsequently the debilitating signs, symptoms, and life-limiting sequelae of Fabry disease [3]. Intrafamilial phenotypic variability is common in Fabry disease [4] and other genetic disorders, such as muscular dystrophy [5], making it difficult to provide an accurate prognosis to patients based only on family history. Levels of disease substrate have been used as biomarkers in various clinical studies in Fabry disease [6, 7]; however, the correlation of changes in these biomarkers with clinical variables remains limited.

Gastrointestinal signs and symptoms are a prominent and clinically important manifestation of Fabry disease and are reported by at least half of patients [8, 9]. Common gastrointestinal signs and symptoms associated with Fabry disease include diarrhea, nausea, vomiting, abdominal pain, and constipation [10, 11]. Gastrointestinal manifestations of Fabry disease are reported from an early age, and often have profound negative effects on social and economic functioning and quality of life in patients [11, 12].

Migalastat is a pharmacological chaperone designed to bind selectively and reversibly with high affinity to the active sites of certain mutant forms of α-galactosidase (amenable *GLA* mutations) [13, 14]. Chaperoning mutated α-galactosidase A to lysosomes may mimic natural enzyme trafficking, which has been suggested to result in more consistent α-galactosidase A activity than current standard of care enzyme replacement therapy (ERT) [15].

In the phase 3 FACETS trial, which included a 6-month placebo-controlled stage, treatment with migalastat maintained stable renal function, reduced cardiac mass, and reduced the severity of gastrointestinal signs and symptoms (diarrhea, reflux, and indigestion domains) in patients with Fabry disease and amenable mutations [15]. In the phase 3, active-controlled ATTRACT study, migalastat and ERT had similar effects on renal function in patients with Fabry disease and amenable mutations, and cardiac mass decreased significantly with migalastat treatment (compared with no change with ERT); furthermore, migalastat was generally safe and well-tolerated [16]. These results led to the approval of migalastat in the European Union, Switzerland, Canada, Australia, Republic of Korea, Japan, and Israel for the treatment of Fabry disease in patients aged 16 years and older with amenable mutations [14, 17].

We report here the results of further analyses using minimal clinically important difference (MCID) to evaluate improvements in diarrhea using the patient-reported Gastrointestinal Symptom Rating Scale (GSRS), in patients with Fabry disease treated with migalastat in the FACETS study. We also examine whether reductions in kidney peritubular capillary (PTC) GL-3 or lyso-Gb_3_ can be used as a surrogate endpoint to predict clinical benefit with migalastat.

## Methods

### Study design and patients

The FACETS trial (AT1001–011, NCT00925301) has been described previously [15]. In brief, the study consisted of a 6-month randomized, double-blind, placebo-controlled phase, followed by a 6-month open-label phase with cross-over of placebo-treated patients to migalastat, and a 12-month extension phase. Male and female patients aged 16 to 74 years with Fabry disease, who were naive to ERT or had not received ERT for at least 6 months before screening, were eligible for randomization [15]. The effect of migalastat on gastrointestinal symptoms was evaluated in patients with *GLA* mutations amenable to migalastat (*N* = 50) [13].

### Gastrointestinal symptoms rating scale

The GSRS comprises 15 questions that assess the severity of 5 domains: diarrhea (“GSRS-D”), abdominal pain, constipation, indigestion, and reflux. Each domain consists of 2–4 questions, each rated on a 7-point Likert scale (from 1—absence of burden to 7—very severe discomfort) [18]. The GSRS-D has 3 questions to assess diarrhea frequency, consistency, and urgency; scores were determined by calculating the mean of the items within this domain. Results were collected at baseline and months 6, 12, 18, and 24 for all patients with amenable mutations, and for the subset of patients presenting with gastrointestinal signs and symptoms at baseline.

### GL-3 levels in kidney peritubular capillaries

Detailed methodology and results of the qualitative assessments of kidney biopsies have been reported [15, 19]. Briefly, kidney biopsies were performed at baseline and at months 6 and 12; these were assessed by 3 independent pathologists using whole slide images at 100× magnification in at least 300 peritubular capillaries in each biopsy to quantify the average number of GL-3 inclusions per PTC. Response to treatment was defined as a reduction of > 0.1 inclusions per capillary (which is above the level of background staining).

### Plasma lyso-Gb_3_

Plasma lyso-Gb_3_ levels were assessed at baseline and at months 6 and 12, and analyzed by means of liquid chromatography-mass spectroscopy [15]. The liquid chromatography-mass spectroscopy plasma lyso-Gb_3_ method used a novel stable isotope-labeled internal standard, ^13^C_6_-lyso-Gb_3_ (lower-limit-of-quantification: 0.200 ng/mL, 0.254 nmol/L) [20, 21]. Response to treatment was defined as any reduction from baseline.

### Statistical analyses

The mean change in GSRS scores from baseline to month 6 was a pre-specified endpoint in the FACETS study. Change from baseline was presented descriptively; statistical tests of significance were performed using an ANCOVA model that included treatment, baseline, and treatment-by-baseline interaction. The *p*-value was calculated based on the comparison of the least squares means.

A response in the GSRS-D was defined as a reduction of 0.33 from baseline (i.e., MCID). The MCID was based on estimates in the literature for several non-Fabry gastrointestinal disorders in which diarrhea is a prominent symptom, and is consistent with an estimate of MCID based on data in Fabry patients from the FACETS study. Specifically, the MCID of 0.33 was derived from anchor-based methodologies from liver transplant patients with gastrointestinal symptoms (MCID = 0.33) [22], patients with autoimmune disease with and without gastrointestinal symptoms (MCID = 0.33) [23], and renal transplant patients with and without gastrointestinal symptoms (MCID = 0.40) [24]. A distribution-based estimate of MCID in Fabry disease was derived from the change from baseline data in the placebo arm of the FACETS study. Using this approach, an MCID of 0.35 was generated, based on half the standard deviation [22, 23], supporting an MCID for GSRS-D of 0.33 in Fabry patients. A sensitivity analysis using a higher threshold of 0.66 was also performed to confirm the results.

The number of patients demonstrating a response in GSRS-D and/or PTC GL-3 from baseline to month 6 was compared between treatment groups. A retrospective analysis using Xu’s statistic, a multivariate test used to assess if treatment has a beneficial effect on multiple outcomes simultaneously [25], evaluated whether treatment impacted both parameters as a combined endpoint. Logistic regression was used to assess the correlation between changes in GSRS-D and PTC GL-3. A similar regression analysis was performed to assess the correlation between changes in GSRS-D and plasma lyso-Gb_3_.

Pre-specified analyses were conducted for all patients with amenable mutations and, post hoc, for the subset of patients with amenable mutations who reported diarrhea symptoms at baseline.

## Results

### Summary of GSRS findings

Of the 50 patients with Fabry disease and amenable mutations who were enrolled in the FACETS study, 28 (56%) reported diarrhea symptoms at baseline.

As previously reported [15], in patients randomly assigned to migalastat (*n* = 28), symptoms of diarrhea, based on the GSRS-D, improved within the first 6 months of treatment (change from baseline, − 0.3), whereas diarrhea in the placebo-treated group (*n* = 22) worsened (change from baseline, + 0.2; *p* = .03). A numerically larger reduction in GSRS-D scores was also observed in the subgroup of patients who reported gastrointestinal symptoms at baseline (migalastat change from baseline, − 0.6; placebo change from baseline, + 0.2). These improvements continued through 24 months of treatment [15].

### Minimal clinically important difference analysis

**Fig. 1:**
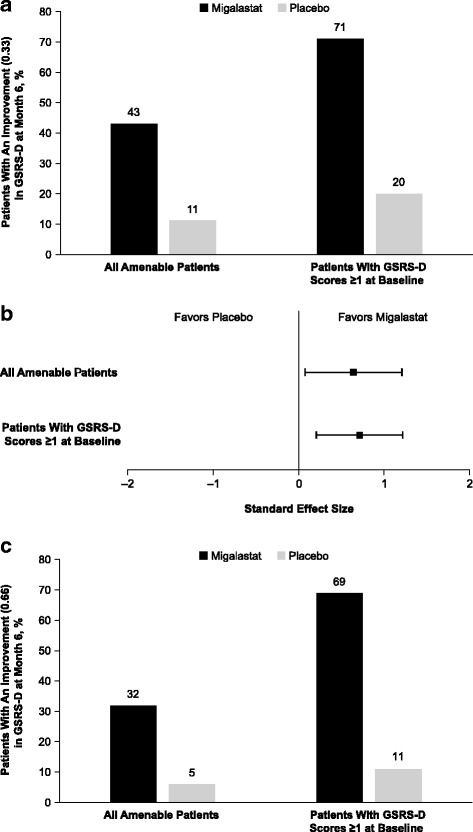
Patients experiencing a minimal clinically important difference in GSRS-D scores after 6 months of treatment. **a** Improvement of 0.33. **b** Forest plot showing the effect size of migalastat treatment vs placebo. **c** Sensitivity analysis: improvement of 0.66

After 6 months of treatment, 12/28 (43%) migalastat-treated patients experienced a GSRS-D score improvement of 0.33 (i.e., MCID) compared with 2/19 (11%) patients receiving placebo (*p* = .02) (Fig. 1a, b). In the subset of patients with diarrhea symptoms at baseline (baseline GSRS-D scores ≥1), 12/17 (71%) of the migalastat-treated patients experienced a clinically relevant improvement of 0.33 compared with 2/10 (20%) of placebo-treated patients (*p* = .02) (Fig. 1a, b).

### Sensitivity analyses

The results of the MCID analysis for observed improvement in diarrhea were confirmed in a sensitivity analysis. Using an improvement threshold of 0.66, 9/28 (32%) migalastat-treated patients experienced a clinically relevant change compared with 1/19 (5%) placebo-treated patients (*p* = .03). In patients with diarrhea symptoms at baseline (baseline GSRS-D score of ≥1), 9/13 (69%) migalastat-treated patients experienced a clinically relevant change compared with 1/9 (11%) placebo-treated patients (*p* = .01) (Fig. 1c).

### GSRS-D and kidney GL-3 inclusions

As previously reported, 6 months of migalastat treatment was associated with a significantly greater reduction in the mean number of GL-3 inclusions per PTC compared with placebo (− 0.25 vs + 0.07; *p* = .008) [15]. An analysis conducted on a combined endpoint of mean change from baseline in PTC GL-3 inclusions and GSRS-D using Xu’s statistic demonstrated a significant treatment effect of migalastat versus placebo (1-sided; *p* = .009).

Assessment of patient-level responses demonstrated a consistent beneficial effect of migalastat on PTC GL-3 inclusions and GSRS-D. A majority of patients with amenable mutations (15/18; 83%) treated with migalastat demonstrated a response in PTC GL-3 and/or GSRS-D when either or both of these endpoints were elevated at baseline, compared with 5/15 (33%) patients treated with placebo.

A logistic regression modeling the improvement in GSRS-D (ie, change from baseline to Month 6 < − 0.33) as the dependent variable, and reduction in PTC GL-3 inclusions (ie, change from baseline <− 0.1, ≥ − 0.1) and treatment group as independent variables, indicated that reductions in PTC GL-3 inclusions were strongly associated with improvement in diarrhea (Table 1). Patients who had a reduction of > 0.1 in PTC GL-3 inclusions (ie, change from baseline <− 0.1) were 5.6 times more likely to have an improvement in diarrhea symptoms than patients who did not have a reduction (*p* = .031).

**Table 1:** Logistic regression assessing correlation between PTC GL-3 reductions and GSRS-D improvement (patients with amenable mutations)

Parameter and Criteria	Odds Ratio	95% CI of Odds Ratio
GSRS-D Reduction From Baseline of 0.33 (*n* = 50)	5.55	(1.17–26.26) *p* = .031
Kidney Peritubular Capillary GL-3 Reduction From Baseline > 0.1		

*CI* confidence interval, *GL-3* globotriaosylceramide, *GSRS-D* Gastrointestinal Symptoms Rating Scale—diarrhea

### GSRS-D and plasma lyso-Gb_3_

When the same correlation analysis was performed for changes in plasma lyso-Gb_3_, defined as any reduction from baseline, and a response in GSRS-D, defined as any reduction ≥0.33, the linear correlation was not statistically significant (odds ratio = 2.5; 95% confidence interval 0.63–9.6; *p* = .2). When the logistic regression was conducted excluding patients with missing data, the odds ratio was similar for PTC GL-3 (odds ratio = 6.6; 95% confidence interval, 1.3–33.0; *p* = .02) and plasma lyso-Gb_3_ (odds ratio = 6.2; 95% confidence interval, 0.6–64.3; *p* = .12); however, statistical significance was achieved only for PTC GL-3, possibly due to the smaller sample size for plasma lyso-Gb_3_ (PTC GL-3, *n* = 44; plasma lyso-Gb_3_, *n* = 31).

## Discussion

In this investigation, migalastat was associated with a clinically relevant improvement in diarrhea symptoms based on the GSRS-D in patients with Fabry disease treated during the phase 3 FACETS trial. Based on the estimated MCID of 0.33, statistically significantly more patients treated with migalastat achieved a clinically meaningful improvement in diarrhea symptoms versus those receiving placebo (43% vs 11%; *p* = .02) after 6 months of treatment. This statistical significance was maintained when the analysis was restricted to only patients with diarrhea symptoms at baseline (71% vs 20%; *p* = .02). A sensitivity analysis using a higher MCID threshold of 0.66 supports these findings. Additionally, a correlation between reduction of PTC GL-3 inclusions and diarrhea improvement was observed; patients with a PTC GL-3 inclusion reduction of > 0.1 were 5.6 times more likely to also have improvement in diarrhea symptoms (*p* = .031). The correlation between reduction in the other disease substrate, plasma lyso-Gb_3_, and improvement in GSRS-D was nonsignificant; a small sample size may have confounded this result.

Diarrhea is among the most common and most troublesome of the gastrointestinal symptoms experienced by Fabry patients. The abnormal accumulation of GL-3 in neurons of the peripheral nervous system, resulting in altered autonomic function and gastrointestinal disturbances, may contribute to the high prevalence of gastrointestinal symptoms in patients with Fabry disease [26, 27]. Since up to 67% of patients report that they experience gastrointestinal symptoms, including diarrhea, some or all of the time [12], and patients report as many as 12 bouts of diarrhea per day [28], symptoms can result in significantly reduced quality of life [11]. Thus, reduction in the severity and frequency of diarrhea can be particularly important for patients. Data from open-label studies with ERT have previously reported improvements in gastrointestinal symptoms following treatment with agalsidase alfa [11] or agalsidase beta [10]. After 12 months of ERT, agalsidase alfa reduced the prevalence of diarrhea by 8% [11]; similarly, following 6–7 months of therapy with agalsidase beta, episodes of diarrhea were reduced, and remained rare or occasional while therapy was maintained (≥3 years) [10]. Our results suggest a similar, clinically relevant improvement in diarrhea symptoms with migalastat treatment.

The analyses presented add to previously published data demonstrating that migalastat improves gastrointestinal signs and symptoms in patients with Fabry disease. In the FACETS study, gastrointestinal signs and symptoms were common, with diarrhea occurring in 56% of patients with amenable mutations at baseline based on the diarrhea domain of the patient-reported GSRS [15]. As previously reported, 6 months of treatment with migalastat resulted in a significant improvement in diarrhea and reflux compared with placebo-treated patients; for diarrhea, this improvement was sustained over 24 months [15]. Over this time, there was also a significant improvement in indigestion and a trend towards improvement in constipation [15].

In the FACETS trial, migalastat reduced substrate levels of GL-3 in patients with Fabry disease [15]. It has been postulated in the literature that GL-3 deposition in endothelial intestinal vasculature and enteric ganglia may contribute to the gastrointestinal manifestations of Fabry disease, with both cell types shown to accumulate GL-3 in Fabry patients with gastrointestinal symptoms [10, 29, 30]. Abnormal function of the enteric plexi is recognized as a potential mechanism causing irritable bowel syndrome, for which patients report similar gastrointestinal symptoms to Fabry disease [28]. Although GL-3 levels in the gastrointestinal tract were not assessed in this study, we hypothesized that other measures of disease substrate (i.e., PTC GL-3 and plasma lyso-Gb_3_) would likely reflect GL-3 changes in the gastrointestinal system, and may be correlated with improvements in gastrointestinal symptoms. Thus, we explored the correlation between reduction in kidney GL-3 inclusions and improvement in GSRS-D scores. The results indicate that reductions in PTC GL-3 inclusions were significantly associated with improvements in diarrhea (GSRS-D scores). Possibly due to the smaller data set available for lyso-Gb_3_, no statistically significant association was observed between plasma lyso-Gb_3_ and GSRS-D scores. Additional studies exploring the potential for plasma lyso-Gb_3_ to be used as a surrogate endpoint are warranted. Based on these results, reductions in GL-3 could be useful as a surrogate endpoint for predicting clinical benefit (i.e., improvement in diarrhea) with migalastat in patients with Fabry disease.

One limitation of these analyses is that the GSRS has not been validated specifically in Fabry disease. Nonetheless, acceptable psychometric properties of the GSRS, including reliability, stability, and construct validity, have been established in patients with irritable bowel syndrome, gastroesophageal reflux disorder, and dyspepsia [18, 31–33]. Across these studies, the GSRS-D has demonstrated consistently strong psychometric properties with reliability (Cronbach’s α) between 0.72–0.84, test-retest stability (intra-class correlation coefficient) between 0.38–0.70, and construct validity, as evidenced by correlations with various health-related quality of life instruments including SF-36, Quality of Life in Reflux and Dyspepsia (QOLRAD), and Psychological General Well-being (PGWB) [32, 33]. These psychometric properties make a case for further use, examination, and perhaps, validation, of the GSRS in the Fabry patient population. An additional limitation is that the reported *p*-values are nominal *p*-values that have not been adjusted for multiplicity. As such, these should be interpreted with caution.

## Conclusions

To our knowledge, the FACETS study is the only double-blind, placebo-controlled study to evaluate gastrointestinal signs and symptoms in patients with Fabry disease. Responder analyses demonstrate that migalastat provided a clinically meaningful reduction in diarrhea in patients with Fabry disease and amenable mutations. These data add to the evidence that migalastat improves gastrointestinal signs and symptoms, including diarrhea, reflux, and indigestion, in patients with Fabry disease [15]. Correlations between GSRS-D scores and PTC GL-3 inclusion reductions suggest that PTC GL-3 inclusions are a potential surrogate endpoint that may predict clinical outcomes with migalastat treatment in Fabry disease. Given that diarrhea occurs frequently and is highly troublesome in Fabry disease, patients with an amenable *GLA* mutation may derive meaningful symptom relief from treatment with migalastat.

## Abbreviations

ERT: Enzyme replacement therapy
GL-3: Globotriaosylceramide
GSRS: Gastrointestinal Symptom Rating Scale
GSRS-D: Gastrointestinal Symptom Rating Scale—diarrhea
lyso-Gb_3_: Globotriaosylsphingosine
MCID: Minimal clinically important difference
PGWB: Psychological General Well-being
PTC: Kidney peritubular capillary
QOLRAD: Quality of Life in Reflux and Dyspepsia
